# Exploring the Bioactive Compounds in Some Apple Vinegar Samples and Their Biological Activities

**DOI:** 10.3390/plants12223850

**Published:** 2023-11-14

**Authors:** Youness El Abdali, Hamza Saghrouchni, Mohammed Kara, Ibrahim Mssillou, Aimad Allali, Yousef A. Bin Jardan, Nesibe Ebru Kafkas, El-Mehdi El-Assri, Hiba-Allah Nafidi, Mohammed Bourhia, Khalid S. Almaary, Noureddine Eloutassi, Abdelhak Bouia

**Affiliations:** 1 Laboratory of Biotechnology, Environment, Agri-Food and Health (LBEAS), Faculty of Sciences Dhar El Mahraz, Sidi Mohamed Ben Abdellah University, Fez 30050, Morocco; 2 Department of Biotechnology, Institute of Natural and Applied Sciences, Çukurova University, Balcalı/Sarıçam, Adana 01330, Turkey; hsaghrouchni@student.cu.edu.tr; 3 Laboratory of Biotechnology, Conservation and Valorisation of Naturals Resources (LBCVNR), Faculty of Sciences Dhar El Mehraz, Sidi Mohamed Ben Abdellah University, Fez 30050, Morocco; mohammed.kara@usmba.ac.ma; 4 Laboratory of Natural Substances, Pharmacology, Environment, Modeling, Health and Quality of Life (SNAMOPEQ), Faculty of Sciences Dhar El Mahraz, Sidi Mohamed Ben Abdellah University, Fez 30050, Morocco; mssillouibrahim@gmail.com; 5 Laboratory of Plant, Animal and Agro-Industry Productions, Faculty of Sciences, University of Ibn Tofail, Kenitra 14000, Morocco; aimad.allali@uit.ac.ma; 6 Department of Pharmaceutics, College of Pharmacy, King Saud University, Riyadh 11451, Saudi Arabia; 7 Department of Horticulture, Faculty of Agriculture, Çukurova University, Balcalı/Sarıçam, Adana 01330, Turkey; 8 Department of Food Science, Faculty of Agricultural and Food Sciences, Laval University, Quebec, QC G1V 0A6, Canada; 9 Department of Chemistry and Biochemistry, Faculty of Medicine and Pharmacy, Ibn Zohr University, Laayoune 70000, Morocco; 10 Department of Botany and Microbiology, College of Science, King Saud University, P.O. Box 2455, Riyadh 11451, Saudi Arabia; 11 Laboratory of Pedagogy and Technological Innovation, Regional Centre of Education and Formation Professions, Fez 30050, Morocco

**Keywords:** apple vinegar, bioactive compounds, antioxidant, antimicrobial, anti-inflammatory, antidepressant

## Abstract

Apple vinegar is highly recommended for nutrition due to its health benefits and bioactive components. However, the apple cultivar greatly influences the quality of the vinegar. In this research, our focus was on examining the impact of four different apple cultivars on the physicochemical attributes, chemical composition, as well as biological properties—including antidepressant and anti-inflammatory activities—of vinegar. Interestingly, the physicochemical properties of vinegar and the contents of acetic acid and polyphenols depend on the apple cultivars. HPLC chromatographic analysis showed that citric acid (820.62–193.63 mg/100 g) and gallic acid (285.70–54.40 µg/g) were mostly abundant in the vinegar samples. The in vivo results showed that administration of Golden Delicious apple vinegar (10 mL/kg) to adult Wistar rats reduced carrageenan-induced inflammation by 37.50%. The same vinegar sample exhibited a significant antidepressant effect by reducing the rats’ immobility time by 31.07% in the forced swimming test. Due to its high acidity, Golden Delicious vinegar was found to be more effective against bacteria, particularly *Bacillus subtilis* and *Candida albicans*, resulting in a MIC value of 31.81 mg/mL. Furthermore, the antioxidant activity of various vinegar samples was found to be powerful, displaying optimal values of IC_50_ = 65.20 mg/mL, 85.83%, and 26.45 AAE/g in the DPPH, β-carotene decolorization and TAC assays, respectively. In conclusion, the apple cultivars used in this study impact the chemical composition and biological activities of vinegar, which may help demonstrate the importance of raw material selection for the production of vinegar.

## 1. Introduction

In the last few decades, attention has been growing regarding the therapeutic application of natural ingredients and food products. In particular, the use of fruit by-products in the food industry as an excellent source of bioactive compounds and nutrients leads to the development of new high-added-value food products [1]. *Malus* species (Apple) belong to the Rosaceae family and are generally considered to be one of the preferred fruits consumed by millions around the globe [2]. Apple vinegar, as a bio-product, constitutes a fresh and nutritious acidic condiment with a distinct flavor and a wide range of health-care benefits. This natural and safe product can be prepared without any additives from various varieties of apple or concentrated apple juice through alcoholic and acetic fermentation, either via a traditional process (Orleans method) or a submerged one (rapid method), which is commonly used in industry [3]. Recently, apple vinegar has become more popular due to consumer needs and interest in functional food quality and due to its wide range of applications [4]. Traditionally used as a food supplement, tonic, and nutraceutical, apple vinegar has also been characterized by several biological properties, including antioxidant, antibacterial, and antifungal activities [3].

Currently, antimicrobial resistance and its related diseases represent a public health challenge. However, natural by-products offer promising bioactivity to circumvent this problem [5]. Therefore, the multiple therapeutic potentials of apple vinegar, especially its antimicrobial activity, offer a safe, natural alternative for cleaning warts, head lice, and nail fungus [3,6] and treating myringitis and otitis [3]. Furthermore, apple vinegar has been shown to be an effective disinfecting agent against SARS-CoV-2, which was responsible for the COVID-19 pandemic [7]. Apple vinegar has also been proven to be an effective product against microbial infections and associated diseases [8]. Additionally, this product has been reported in previous studies as a natural preservative and disinfectant of vegetables and fruits against foodborne pathogens and for the prevention of food spoilage and discoloration [9,10].

Oxidative stress and inflammation have been established to be pivotal for the development and progression of many chronic diseases associated with oxidative stress and inflammation. Notably, the regulation of oxidative stress and the inflammatory response is very important to the maintenance of the internal function of the human body, and any imbalance can lead to certain chronic diseases, such as cardiovascular and neurodegenerative diseases and even cancer [11,12]. Nowadays, the search for natural antioxidant and anti-inflammatory agents is necessary to tackle oxidative stress and inflammation [6]. According to several studies, apple vinegar is an excellent antioxidant and considered a good free radical scavenger, which can limit or reduce oxidative stress and therefore reduce inflammation [13,14]. Moreover, apple vinegar is characterized by many other pharmacological effects; it has been reported as an antidiabetic [15], antihypertensive [6], and anti-Alzheimer agent [16]. In addition, the consumption of apple vinegar prevents weight gain, hyperlipidemia, and hypercholesterolemia and improves glucose tolerance [6,15]. These properties promote the use of apple vinegar as a good alternative to chemical conservatives and synthetic products that induce negative effects on human health.

All the mentioned health-protective effects of apple vinegar are correlated to its chemical composition [17]. Certainly, apple vinegar is an inexhaustible source of several bioactive compounds, such as carbohydrates, organic acids, flavonoids, polyphenols, vitamins, and minerals [3,17]. Many factors can influence the quality and functional properties of apple vinegar, including storage conditions, storage time, and production techniques [3]. However, the variety used in the preparation of the vinegar and its stage of maturity are the main factors that influence the composition and biological properties of apple vinegar, as reported by several studies [13,14].

To our knowledge, few studies have evaluated the anti-inflammatory and antidepressant activities of apple vinegar. Therefore, considering the effects of the raw material on the composition and quality of apple vinegar, this study was designed to assess and characterize the impact of varietal profiles on the physicochemical characteristics; bioactive compounds; and antioxidant, antimicrobial, anti-inflammatory, and antidepressant properties of vinegar produced from four apple cultivars (Golden Delicious, Red Delicious, Starking Delicious, and Gala) in the region of Imouzzer Kander (Morocco).

## 2. Results and Discussion

### 2.1. Physicochemical Properties and Total Phenolic and Flavonoid Content

The physicochemical properties of each vinegar sample are presented in Table 1. The parameters studied offer relevant information on the quality of the vinegar. In this context, small differences were observed in the pH values, which range from 2.84 ± 0.01 to 3.82 ± 0.04, while the lowest value was recorded in the vinegar produced from the Gala cultivar. The density values of the vinegars were close and ranged between 0.962 and 1.018 g/cm^3^. The vinegar samples studied allowed the passage of electric current; thus, the electrical conductivity values obtained ranged from 2.96 ± 0.02 mS/cm in Gala to 3.48 ± 0.02 mS/cm in Red Delicious. Generally, conductivity is relative to the content of suspended mineral matter in the medium [18]. The quantity (in grams) of acetic acid equivalent to the total acidity in 100 mL of vinegar at 20 °C was used to determine the average titratable acidity (%). The analyzed vinegar showed different total acidity values. Golden Delicious vinegar contained the highest acetic acid content of 2.56 ± 0.14% compared to Starking Delicious vinegar (0.28 ± 0.06%). Indeed, the acidity of the vinegar is closely related to the sugar content of the apple, which directly influences the ethanol content and, consequently, the acetic acid content of the vinegar obtained after the alcoholic and acetic fermentations. In this context, several studies have reported a high sugar content in the Golden Delicious apple variety compared to the Gala and Starking Delicious varieties. This explains the low acidity values observed in Gala and Starking Delicious vinegar compared to Golden Delicious vinegar [19,20]. In addition, the degree of ripeness of the apple fruit also affects the sugar and organic acid content in vinegar, which can have an impact on the microbiological stability and the flavor, and, consequently, the acidity and the pH of the vinegar [3,14]. In general, the results of the physicochemical parameters of our samples are approximatively within the range of other investigations [3,13,17,21]. In addition to the physicochemical properties, the varieties of vinegar showed a variable amount of total phenols. Phenolic content values ranged from 3.72 ± 0.15 mg GAE/g (Golden Delicious) to 6.12 ± 0.02 mg GAE/g (Starking Delicious). Otherwise, no significant difference (*p* > 0.05) was observed in the flavonoid content of these samples, and the values ranged between 0.254 ± 0.008 mg and 0.296 ± 0.012 mg QE/g. These differences in the content of total phenolic compounds and flavonoids were also observed in samples of vinegar from three apple varieties from different regions of Morocco. Reported values ranged from 68.91 ± 4.50 to 147.54 ± 12.10 mg GAE/100 mL for total phenols and between 4.72 ± 0.20 and 15.32 ± 0.20 mg QE/100 mL for flavonoids [13]. Variability in total phenolic and flavonoid contents was also noted in a Turkish study conducted on twenty traditional home-made vinegars, six of which were made from apple fruit [17]. The raw material and the production method of the vinegar affect the total phenolic content. Moreover, the ripening stage of apples, the cultivation conditions, and genetic as well as climatic interactions may also intervene [14,22]. The enzymatic transformation during fermentation processes can also influence the phenolic content of vinegar [23]. Overall, a wide variability in the physicochemical characteristics and bioactive phytochemicals of our vinegar samples was detected, reflecting remarkable differences in the quality of the vinegar. Nevertheless, this variability mainly seems related to the type of apple cultivars used in the preparation of vinegar.

### 2.2. Organic Acid and Polyphenol Composition

**Figure 1:**
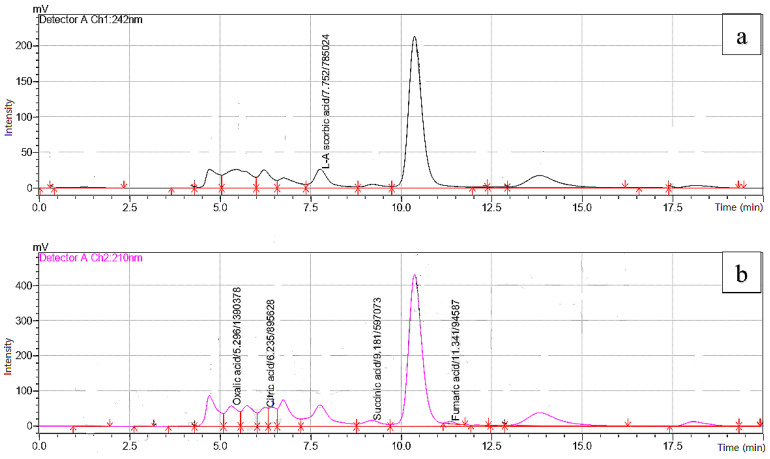
Chromatographic profile of organic acids detected at 242 nm (**a**) and 210 nm (**b**) in Golden Delicious apple vinegar.

The organic acids identified in the apple vinegar studied and their contents are listed in Table 2. Retention times, UV spectra, and peak areas were used to identify the organic acids and quantify their content (Figure 1). Among the five organic acids detected, malic acid was the major organic acid in Golden Delicious vinegar (241.10 mg/100 g), while citric acid was the most abundant in all other vinegar samples, where the highest content (820.62 mg/100 g) was recorded in Red Delicious vinegar. Moreover, the same sample contained the highest amount of malic acid (280.16 mg/100 g) and oxalic acid (45.92 mg/100 g). Ascorbic acid was mainly found in Golden Delicious vinegar, with a concentration of 15.78 mg/100 g. The highest concentration of fumaric acid was detected in Gala vinegar (67.32 mg/100 g). The main organic acids identified in the Starking Delicious vinegar were, respectively, citric acid (532.86 mg/100 g) and malic acid (174.57 mg/100 g). These compounds have been previously reported in a study conducted on traditional Turkish apple vinegar, where acetic (84.2%), succinic (6.3%), and lactic (5.2%) acids have been the most abundant [24]. Acetic acid (29.8%) has also been reported as the most abundant acid among the eight organic acids detected in six traditional homemade apple tablespoons of vinegar previously studied [17]. Similarly, malic acid (7691.98 µg/mL) followed by citric acid (6485.24 µg/mL) and lactic acid (2541.64 µg/mL) have been the main organic acids found in apple vinegar purchased from a local market in China [4]. The findings of the same study confirmed that the apple variety plays a determining role in terms of the concentration and type of organic acids present in apple vinegar. Indeed, the concentration and types of organic acids affect the sensory and organoleptic quality of apple vinegar, as well as their health functions. These compounds are produced throughout the fermentation process by hydrolysis, microbial activity, and biochemical metabolism [4]. Therefore, it has been proved that the concentration and type of organic acids in apple cider and vinegar depend on the yeast involved in the fermentation process, especially non-Saccharomyces yeasts and probiotics [22,25]. Organic acids are important bioactive components of vinegar that exert many health benefits by regulating lipid abnormalities, controlling blood glucose [26], and possessing significant antimicrobial activity [27].

**Figure 2:**
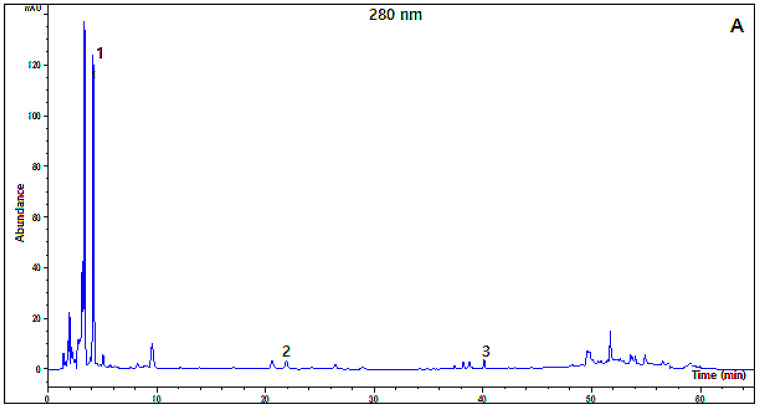

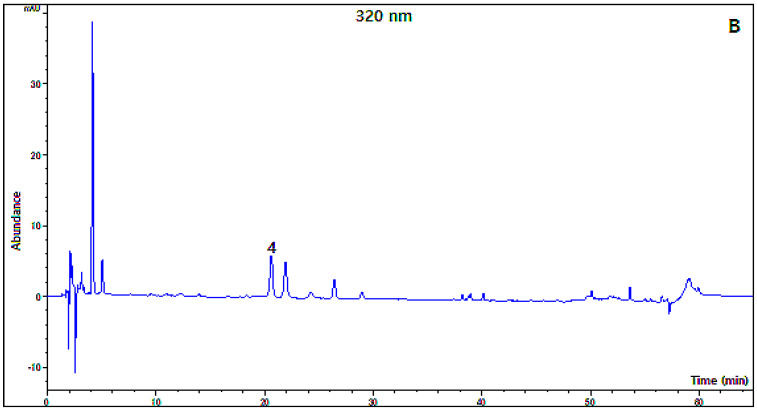
Chromatographic profile of polyphenolic compounds detected at 280 nm (**A**) and 320 nm (**B**) in Golden Delicious apple vinegar. Gallic acid (peak 1); chlorogenic acid (peak 2); myricetin (peak 3); caffeic acid (peak 4).

The polyphenolic compounds of the apple vinegar studied were also analyzed. Figure 2 and Table 2 represent, respectively, the HPLC chromatographic profile of Golden Delicious apple vinegar and the content of compounds identified in the samples. As listed in Table 2, a total of five phenolic compounds were identified. Gallic acid was the major phenolic compound present in all varieties of vinegar, with contents ranging between 54.40 µg/g in Golden Delicious vinegar and 285.70 µg/g in Gala vinegar. However, the results revealed the presence of chlorogenic acid (2.65 µg/g) and naringenin (1.84 µg/g) only in Golden Delicious and Starking Delicious vinegar, respectively. Additionally, myricetin and caffeic acid were identified in Red Delicious and Golden Delicious apple vinegar, with the highest concentration reaching 22.24 and 6.44 µg/g, respectively, whereas both compounds were not detected in the other vinegar samples. Our results are in accordance with those reported by Liu et al. (2019). This study reported the presence of protocatechuic acid (0.08–1.54 µg/mL), chlorogenic acid (0.11–10.91 µg/mL), and p-coumaric acid (0.10–0.17 µg/mL) as the main phenolic compounds in fourteen apple vinegar purchased from a local market in China [4]. The authors concluded that the different varieties of apples used to produce the vinegar were the main reason for the variation in the phenolic composition of the samples. Similar results have also been reported in traditional Turkish apple vinegar, where gallic acid (4.5 µg/mL), catechin (1.3 µg/mL), protocatechuic acid (0.7 µg/mL), and caffeic acid (0.3 µg/mL) have been the main phenolic compounds detected [24]. In fact, the phenolic compounds found in the vinegar studied were mainly derived from the raw materials used in their production. However, other factors may influence the phenolic composition of vinegar. According to the results of a study on cider vinegar produced with three varieties of apples at different stages of ripeness, the genetic variability between these varieties, and the degree of ripeness, have been the main factors influencing the concentration and nature of phenolic compounds present in the final vinegar product [14]. The authors explained the increase in phenolic compounds observed between the ripe and senescent stages by the high activity of pectinolytic enzymes in the fruit, which facilitates extraction during vinegar processing. Moreover, the findings of the same study revealed significant changes in the polyphenolic composition and their concentrations during the alcoholic and acetic fermentation of apple cider vinegar. This partly explains the qualitative and quantitative differences in the polyphenolic composition found in our vinegar samples. The therapeutic properties and health benefits of apple vinegar are strongly linked to its phenolic compounds. In addition to their antioxidant potential, these dietary bioactive molecules play a crucial role in the antimicrobial, anti-inflammatory, anti-cancerous, and antidepressant properties of apple vinegar [28,29,30]. Furthermore, polyphenolic compounds are mainly involved in the quality of the vinegar, its color, as well as its astringency [28]. Overall, our results indicate that the apple vinegar studied had an abundance of organic acids and polyphenolic compounds with diverse and complex compositions. The different apple cultivars used to prepare the vinegar seem to be the main factor implicated in this variability.

### 2.3. Antioxidant Capacity

**Figure 3:**
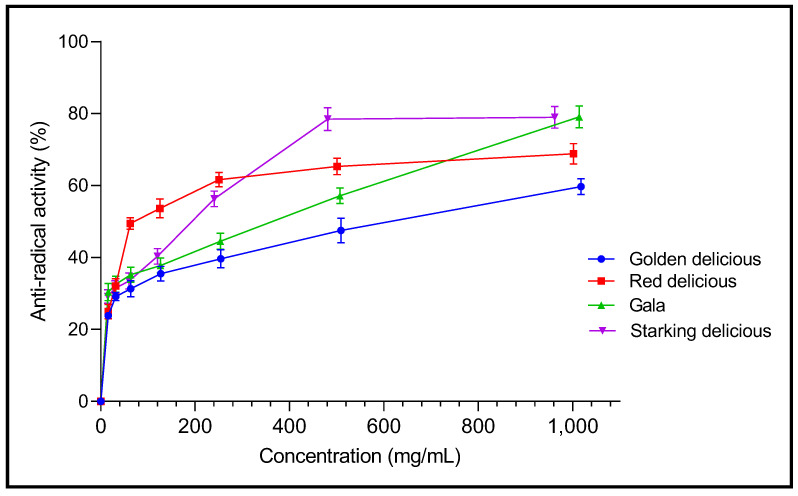
DPPH free radical scavenging activity of the studied vinegars.

A multi-test approach was carried out to evaluate the antioxidant properties of the vinegars studied. The results are presented in Table 3. The antioxidant activity of the samples varied depending on the test performed and the apple cultivar used in the vinegar processing. Using the DPPH assay, the four varieties exhibited various anti-free radical abilities in a dose-dependent manner (Figure 3). The anti-radical activity of the Gala and Starking Delicious vinegar reaches a maximum of 79.09% and 78.96%, respectively, which is higher than the other vinegar. Calculated IC_50_ values ranked the studied vinegar in terms of DPPH free radical scavenging. Red Delicious, followed by Starking Delicious vinegars, displayed stronger antioxidant activity, with the following lowest IC_50_ values: 65.20 ± 13.00 and 187.70 ± 18.10 mg/mL, respectively. In contrast, Golden Delicious vinegar exhibited the weakest DPPH anti-free radical activity by recording the highest IC_50_ value (596.00 ± 29.50 mg/mL). Compared to the vinegar samples, the BHT standard recorded a significantly lower (*p* < 0.05) IC_50_ value of 0.15 ± 0.04 mg/mL.

Fatty acid degradation is one of the primary causes of food spoilage, as reported in several studies. To limit food spoilage, vinegar has been frequently used in industry as a food preservative. In this research, antioxidant activity was assessed by measuring the ability of apple vinegar to inhibit linoleic acid oxidation using β-carotene as a marker. As shown in Table 3, all apple vinegar samples possessed the ability to limit fatty acid peroxidation, with varying inhibition rates. Gala vinegar exhibited a powerful antioxidant effect by limiting a maximum of 85.83 ± 2.13% oxidation of linoleic acid compared to other vinegar. The lowest scavenging activity was noted in Starking Delicious vinegar, which inhibited only 69.17 ± 2.03% of β-carotene discoloration. ANOVA analysis showed no significant difference between the antiradical activity of Gala vinegar and BHT (*p* > 0.05).

The total antioxidant capacity (TAC) of the vinegar samples was also assessed using a phosphomolybdenum assay. Regarding the results obtained (Table 3), the total antioxidant capacity of the vinegar varied between a minimum value of 16.58 ± 0.18 mg AAE/g in Gala vinegar and a maximum value of 26.45 ± 0.82 mg AAE/g in Golden Delicious vinegar. Compared to our vinegar samples, the reference antioxidants BHT (46.87 ± 0.72 mg AAE/g) and quercetin (28.06 ± 0.29 mg AAE/g) were significantly superior in their total antioxidant capacity (*p* < 0.05).

Many researchers have confirmed and demonstrated the antioxidant property of apple vinegar. Ousaaid and his team evaluated the antioxidant activity of Moroccan vinegar produced from various apple varieties in four different bioclimatic zones using DPPH free radical scavenging and TAC assays. Significant DPPH anti-free radical activity has been detected in the indicated study, with IC_50_ values ranging between 0.45 ± 0.013 and 1.19 ± 0.014 µL/mL. Similarly, the total antioxidant capacity in the same study varied between 2.29 ± 0.58 mg and 13.27 ± 0.47 mg AAE/100 mL [13]. This variability in free radical scavenging activity has also been reported in another study conducted on six traditional homemade apple vinegars from Turkey. The results showed that the DPPH free radical scavenging values ranged from 0.53% to 65.12% [17].

Besides the raw material, the degree of ripeness of the apples also influences the antioxidant activity of the vinegar. This finding has been reported in a study conducted on three table apple varieties (Fuji Suprema, Gala, and Lis Gala) manipulated at three different ripening stages (unripe, ripe, and senescent) using DPPH and FRAP assays [14]. In general, the antioxidant propriety is related to the phenolic and organic acid compounds present in the samples [4]. Many studies demonstrated the links between phenolic compounds and the antioxidant activity of vinegar. For instance, a high correlation has been observed between the phenolic content (including all phenolic classes) and antioxidant activity of apple vinegar using FRAP (r = 0.89, *p* < 0.001) and DPPH (r = −0.97, *p* < 0.001) assays in the study conducted by [14]. However, the correlation remains non-significant between the DPPH (IC_50_%) and the polyphenolic and flavonoid compounds of apple vinegar (r = −0.033048; *p* > 0.05 and r = −0.58876; *p* > 0.05, resp.), as reported in other investigations [13,17].

Moreover, hydroxycinnamic acids, gallic acid, *p*-coumaric acid, (+)-catechin, and (−)-epicatechin polymers (procyanidins) also appear to be involved in the antioxidant activity of apple vinegar, as previously reported [4,14]. The antioxidant effect of apple vinegar may also be linked to the presence of potent antioxidants such as phloretin and quercetin, which both increase during the fermentation process [22]. Furthermore, an in vivo study demonstrated that consumption of apple vinegar effectively improved oxidative stress in rats by increasing the activities of critical enzymes that defend against oxidative stress (glutathione peroxidase, catalase, and superoxide dismutase) from 15 to 66% compared to control rats [31]. In summary, the results obtained show that the selection of the raw material during the preparation of the vinegar plays a determining role in the antioxidant activity and, consequently, the quality of the final product.

### 2.4. Antimicrobial Activity

The antimicrobial activity of the four apple vinegar samples was evaluated in vitro—qualitatively (Table 4) and quantitatively (Table 5)—against bacterial and fungal strains using two methods, including agar diffusion and microdilution tests. The three bacterial strains (*B. subtilis*, *S. aureus*, and *E. coli*), in addition to *C. albicans* tested in the present study, are among the most frequent microbes to develop multidrug resistance and are considered acquired infections both in the community and hospitals [32].

Table 4 represents the results of the inhibition diameters in mm caused by the diffusion in the agar medium. The largest inhibition diameters were given by Golden Delicious vinegar, where the diameters were 25, 15, 14.5, and 11.5 mm against *B. subtilis, E. coli*, *S. aureus,* and *C. albicans*, respectively. Next is Gala vinegar, with a diameter of 17 mm against *B. subtilis*. Finally, Red Delicious and Starking Delicious were less active, where a diameter of 12 mm was observed against *E. coli* and *B. subtilis*, respectively.

Table 5 describes the results of the MIC values of different vinegar samples. Golden Delicious vinegar revealed the highest antimicrobial activity, where a MIC value of 31.81 mg/mL was observed against *B. subtilis, S. aureus,* and *C. albicans.* Red Delicious vinegar comes next, with a MIC value of 62.62 mg/mL against *E. coli*, *B. subtilis,* and *S. aureus.* Regarding the effect of the Gala and Starking Delicious vinegar, the MICs vary between 126.75 and 481 mg/mL.

According to the results, Golden Delicious vinegar was more active against *C. albicans* and Gram-negative and -positive bacteria, followed by Red Delicious vinegar, which was more active against all bacterial strains, However, against *C. albicans,* no important effect was revealed. In addition, the Gala and Starking Delicious vinegars were only active against *B. subtilis.*

In a recent study [13], the antimicrobial effect of seven samples of vinegar collected from different regions of Morocco was investigated against *S. aureus* and *E. coli*. Therefore, the diameter inhibition ranged from 14.85 to 27.65 mm and from 11.05 to 20.5 mm against *S. aureus* and *E. coli*, respectively. Moreover, MICs ranged from 1.125 to 25.00 mg/mL and 0.625 to 25.00 mg/mL against *S. aureus* and *E. coli*, respectively. Of these seven vinegar samples, one showed no effect [13]. A similar study conducted on four varieties of apple vinegar in the same country demonstrated that Red Delicious was the most active vinegar against *S. aureus* and *E. coli*, recording inhibition diameters of 19 and 15 mm, and values of MICs reached, respectively, 7.81 and 1.95 µL/mL [3]. In another study carried out in Turkey, six vinegar samples were collected from different cities [17]. Antimicrobial activity was evaluated by only using the agar diffusion method. Therefore, a single sample showed the highest antibacterial activity against *B. cereus* and *S. aureus,* with a zone of inhibition reaching 23.56 and 20.12 mm, respectively. In contrast, against *E. coli*, an inhibition of 10.75 mm was revealed, and the rest of the samples showed no activity [17].

Among the microbial strains tested, *B. subtilis* and *S. aureus,* which are Gram-positive bacteria, were the most sensitive, followed by *E. coli*, and the least sensitive was *C. albicans*. These results are in accordance with other previous reports that have shown the sensitivity of Gram-positive bacteria, and especially *S. aureus*, to vinegar as well as its sensitivity to most bioactive compounds [6,33]. Indeed, this sensitivity has been linked to the permeability of the cell wall of Gram-positive bacteria compared to the membrane of Gram-negative bacteria, which contains two membranes that serve as a barrier against antibacterial compounds [34].

Vinegar with high acidity due to the presence of organic acids, including citric, malic, tartaric, lactic, acetic, and succinic acids, can inhibit microbial growth depending on their level of acidity [24,35]. Weak acids like acetic acid show their antimicrobial activity by crossing the microbial membrane, thereby decreasing the intracellular pH, which leads to the release of protons into the cytoplasm and the death of microorganisms [35,36,37]. Moreover, organic acids have been shown to disrupt the outer membrane of bacteria, inhibit microbial enzymes, block energy utilization and macromolecular synthesis, and increase the intracellular osmotic pressure of pathogens [38,39]. Apart from organic acids, vinegar also contains considerable amounts of phenolic molecules, such as gallic, protocatechuic, chlorogenic, and caffeic acids, which are known to possess strong antimicrobial activity against bacteria [39,40,41] and fungi [40,42]. In this study, all apple vinegar samples examined contained considerable levels of phenolic compounds and organic acids, which may be responsible for the documented antibacterial effect.

### 2.5. Anti-Inflammatory Activity

**Figure 4:**
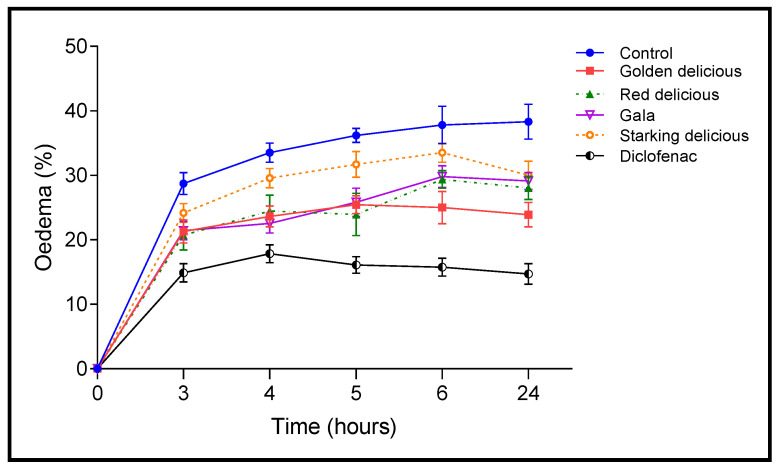
Paw edema (%) of rats treated with the vinegars and diclofenac after 3 h, 4 h, 5 h, 6 h, and 24 h of carrageenan injection.

**Figure 5:**
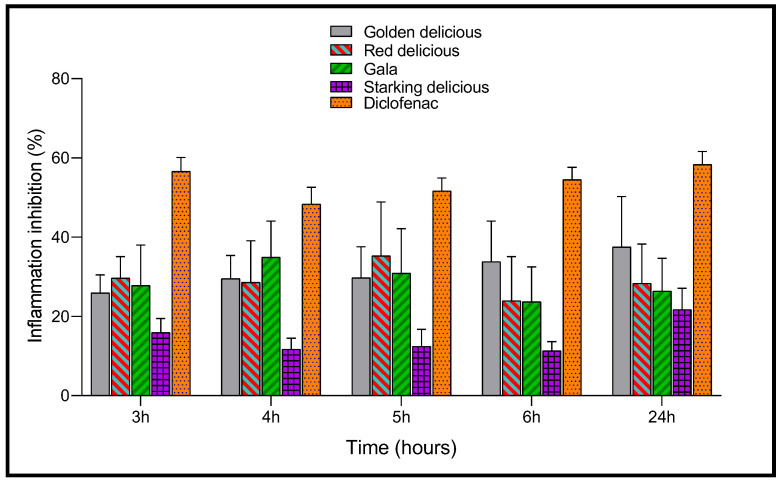
Effect of vinegars and diclofenac administered orally on carrageenan-induced edema in rats. Data are expressed as means ± SEM; *p* < 0.05 is considered significant relative to the control.

The anti-inflammatory activity of the vinegars studied (10 mL/Kg) was tested by the rat paw test induced by carrageenan. Rat paw circumference (*n* = 5), as well as inflammation inhibition (%) at 3, 4, 5, 6, and 24 h after carrageenan (1%) injection, are, respectively, shown in Figure 4 and Figure 5. During the first four hours, an increase in edema (volume of the paws) was observed for all the rats tested. After 24 h, the paw edema of the rat paw showed the highest level (38.30 ± 3.96%) in the control. In comparison, the increase in paw edema was lower in all rats treated with the vinegar samples studied throughout the test period. For example, after 4 h of carrageenan injection, the vehicle (0.9% NaCl) displayed the highest level of edema (33.51 ± 3.44%). Regarding the other groups treated with Starking Delicious vinegar (29.57 ± 2.22%), Red Delicious vinegar (24.46 ± 4.89%), Golden Delicious vinegar (23.62 ± 2.33%), Gala vinegar (22.53 ± 3.08%), and diclofenac (17.85 ± 0.17%), it can be seen that all the vinegars tested showed an anti-inflammatory effect. After 24 h, all vinegar samples exhibited a significant reduction in paw edema at *p* < 0.05 compared to the control (Figure 4).

Additionally, Figure 5 illustrates that after 24 h, diclofenac (1%) presented the highest inhibition of inflammation (58.30%), followed by Golden Delicious vinegar (37.50%), Red Delicious vinegar (28.30%), and Starking Delicious vinegar, the latter of which showed only 21.67% inhibition of edema.

The carrageenan-induced rat paw inflammation assay provides a suitable in vivo animal model to examine the anti-inflammatory activity of multiple natural products [43]. The inflammation is caused by the metabolism of arachidonic acid, which leads to increased tissue fluid and extravasation of neutrophils and plasma [44]. Carrageenan causes the release of mediators such as bradykinin, histamine, and serotonin in the early phase of hyperemia. After 2–3 h of carrageenan injection, paw edema reaches its peak and then begins to decline [45]. The second phase is defined by the release of prostaglandin, followed by the migration of leukocytes into the inflammatory site [46].

Our results show that oral administration of the tested vinegar reduces the edematous volume of the rat paw from the 3rd hour of inflammation, suggesting that the significant inhibitory activity of the 2nd inflammatory phase specifically targeted the prostaglandin mediator through the cyclooxygenase pathway. In this context, significant inhibition of the activity of cyclooxygenase-1 by acetic acid (the main organic acid of apple vinegar) has been experimentally proven; consequently, it has an influence on the disruption of the synthesis of prostaglandin via the arachidonic acid pathway [47]. Moreover, the anti-inflammatory activity of apple vinegar and acetic acid and its inhibitory effects on pro-inflammatory cytokines such as TNFα and IL-6 have also been proven by various anti-inflammatory tests in vivo and in vitro, [6,48]. Further mechanistic investigation showed that vinegar and acetic acid reduce ulcerative colitis inflammation in mice by inhibiting Th1 and Th17 responses, NLRP3 inflammasome, and activation of MAPK signaling [49].

It is necessary to mention that apple phenols are also involved in the downregulation of prostaglandin and cytokines (TNF and IL-6) in the case of gastrointestinal cell inflammation [29]. Polyphenols have also been associated with the signaling process of pathways modulating gene expression that enhance an anti-inflammatory environment [50]. Furthermore, the hydroxyl group of many flavonoids has been shown to impact oral anti-inflammatory activity [51]. The results obtained give the studied vinegars an anti-inflammatory mechanism of action similar to that of non-steroidal anti-inflammatory drugs and could be explained by the presence of bioactive compounds such as phenolic compounds and organic acids.

### 2.6. Antidepressant Activity

**Figure 6:**
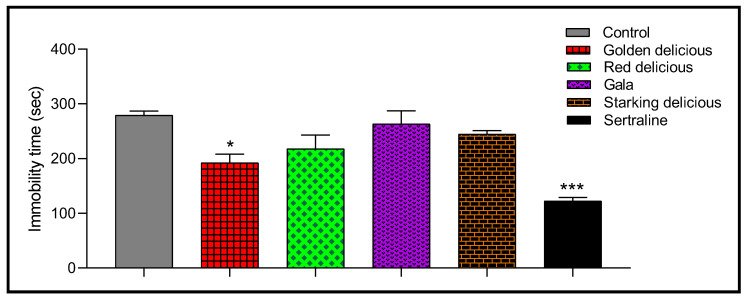
Antidepressant effect of vinegar and sertraline in rats (means ± SEM). * *p* < 0.05 and *** *p* < 0.01 are considered significant relative to the control.

The forced swimming test, as a model of depression, was performed to evaluate the antidepressant activity of vinegar samples, and the results are shown in Figure 6. The immobility time of the control group was 279.0 ± 7.75 s for 6 min. Oral administration of Golden Delicious vinegar produced significant antidepressant activity in rats (192.25 ± 15.88 s). This effect resulted in a reduction in the immobility time of 31.07% compared to the control lot (*p* < 0.05). On the other hand, no statistically significant antidepressant effect was noted in the other vinegars (*p* > 0.05). With the exception of Golden Delicious vinegar, the effect of all the vinegars was significantly reduced compared to that of sertraline (122.5 ± 6.46 s of immobility time).

The forced swimming test resulted in depression of the rats’ affective state. Several studies have implicated the role of altered serotonergic discharge as one of the primary pathogenesis of depressive disorders [52,53]. Sertraline is a selective serotonin reuptake inhibitor (SSRI) antidepressant, which works by inhibiting the neuronal reuptake of serotonin (5-HT serotonin). As a result, the intracerebral level of this neurotransmitter increases, which alleviates depressive symptoms [54].

The finding that Golden Delicious vinegar alleviated depressive-like behaviors by reducing the time of immobility may suggest its beneficial effect in depression-related conditions through a possible increase in the serotonergic system. The same conclusion has been reported in the study conducted by Asejeje et al., confirming that the administration of apple vinegar reduced attenuated lipopolysaccharide-induced anxiety and depression-like behaviors in mice via possible involvement of the serotonergic system [48]. The same investigation found that apple vinegar effectively prevented a lipopolysaccharide-induced increase in levels of brain proinflammatory cytokines levels (IL-6 and TNF-α) and, consequently, the alleviation of depression-like behaviors in mice.

Apple vinegar is an inexhaustible source of polyphenols and flavonoid compounds. Interestingly, these molecules exhibit significant antidepressant activity, as reported in several studies [50,55]. Practically, these phenolic molecules, such as naringenin and chlorogenic acid found in our vinegar samples, have been reported as modulators of monoaminergic, nor-adrenergic, serotoninergic, and dopaminergic neurotransmission in the brain and consequently exhibited antidepressant-like behaviors in animal models of depression [30]. Other mechanisms supporting the antidepressant potential of polyphenols include their anti-inflammatory potential, antioxidant properties, ability to modulate monoamine oxidase enzyme activity, and actions on the neurotransmitter receptor system [30]. Similarly, the antidepressant effect of flavonoid compounds has been widely confirmed in various human studies and animal models of depression [56,57]. Flavonols, isoflavones, and flavan3-ols have been mentioned as the major flavonoid subclasses involved in an antidepressant activity [56]. Overall, we can suggest that the diversity of polyphenolic and flavonoid chemicals contained in our vinegar samples explains the reported antidepressant activity.

### 2.7. Correlation and Multivariate Statistical Analysis:

Statistical analysis was performed to establish and understand the links between the investigated quality parameters of apple vinegar and their biological activities.

First, Pearson’s correlation was used to calculate the correlation between the quality parameters and biological activities of all vinegar samples, and the results are listed in Table 6. Statistically significant correlations were observed between certain quality characteristics and biological activities. For example, the highest correlation between total phenols and the IC_50_ of DPPH scavenging capacity (r = −0.919) reflects the significant involvement of these chemicals in the scavenging propriety of apple vinegar, as reported in several investigations [4,14]. Interestingly, the type and amount of each phenolic compound contained in the vinegar exhibited different respective activity levels [58]. On the other hand, this difference observed between the results of the different antioxidant activity assays used and their dependence on phenolic compounds is considered to be caused by the variable reaction mechanism used to evaluate the antioxidant effect. Inhibition of lipid oxidation is based on hydrogen atom transfer, while the free radical scavenging ability (DPPH) depends on both the transfer of hydrogen atoms and the transfer of a single electron [59].

Besides the antimicrobial action of the phenolic components of vinegar, a high statistical correlation was found between acetic acid and antimicrobial activity against *C. albicans*, *E. coli,* and *S. aureus* (r = 0.868, r = 0.851 and r = 0.812, respectively). The acetic acid content of vinegar was also significantly correlated with total antioxidant, anti-inflammatory, and antidepressant activities (r = 0.867, r = 0.976, and r = 0.903, respectively). The significant involvement of acetic acid in the activities studied has already been reported [4,24,47]. However, flavonoids appear to be moderately active against *C. albicans* and *B. subtilis* (r = 0.459 and r = 0.508, respectively).

**Figure 7:**
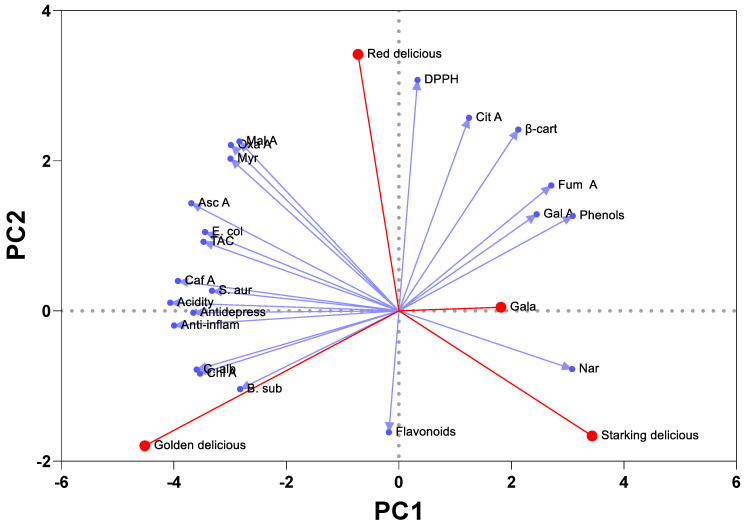
PCA analysis of vinegar using the following tested parameters as input: total phenols; flavonoids; acetic acid % (Acidity); antiradical activity (IC_50_ of DPPH assay); total antioxidant capacity (TAC); β-carotene antiradical activity (β-car); antidepressant activity (Antidepress); anti-inflammatory activity (Anti-inflam); inhibition zone diameters of *C. albicans* (C. alb), *E. coli* (E. col), *B. subtilis* (B. sub), and *S. aureus* (S. aur); ascorbic acid (Asc A); oxalic acid (Oxa A); citric acid (Cit A); malic acid (Mal A); fumaric acid (Fum A); gallic acid (Gal A); caffeic acid (Caf A); chlorogenic acid (Chl A); myricetin (Myr); and Naringenin (Nar).

A principal component analysis (PCA) was also performed to explore the statistical relationship between the assessed variables and the distribution of the vinegar samples (Figure 7). The two main components in Figure 7 successively represented 54.57% and 26.80% of the total information contained in the original data matrix. Analysis of the statistical results revealed a significant correlation between acetic acid content and total antioxidant, antimicrobial, anti-inflammatory, and antidepressant activities. However, the total phenolic content was mainly correlated with the antiradical activity. At the molecular level, chlorogenic and caffeic acids were, statistically, the main polyphenolic compounds involved in the anti-inflammatory and antidepressant activities of the vinegar studied. Additionally, these compounds were also correlated with antimicrobial activity against *B. subtilis, S. aureus*, and *C. albicans*. Gallic acid, which was the main polyphenolic compound of the vinegars studied, was strongly correlated with the free radical scavenging activity (DPPH). PCA analysis results also show that organic acids such as citric and fumaric acids were significantly active in inhibiting lipid oxidation, as measured by the β-carotene assay, whereas ascorbic acid seemed to be responsible for the antibacterial activity against *E. coli*.

The significant involvement of these bioactive molecules in the biological and pharmacological activities studied has already been reported [6,14,24,29,30]. Considering the similarities of the vinegar cultivars studied, PCA made it possible to differentiate three categories. Notably, Golden Delicious vinegar stands out in the first category, showcasing remarkable antimicrobial, anti-inflammatory, and antidepressant potentials attributed to its elevated acetic acid content. The second category encompasses Red Delicious and Gala vinegars, both exhibiting similar properties primarily linked to their phenolic content, notably in inhibiting lipid oxidation, as observed in the β-carotene test. In contrast, Starking Delicious vinegar forms a distinct category, distinguished by its abundant phenolic and flavonoid compounds.

## 3. Material and Methods

### 3.1. Chemicals and Reagents

Folin–Ciocalteu, sodium carbonate, aluminum trichloride, calcium chloride, β-carotene, linoleic acid, Tween 80, hydrogen peroxide, chloroform, 2,2 diphenylpicrylhydrazyl (DPPH), ammonium molybdate, quercetin, butylated hydroxytoluene (BHT), ascorbic acid, sodium phosphate, and sulfuric acid (analytical grade) were obtained from Sigma-Aldrich (Munich, Germany). The microbial growth mediums, antibiotics, and 2,3,5-triphenyltetrazolium chloride (TTC) were purchased from Biokar Diagnostics (Allonne, France). Carrageenan, diclofenac, and sertraline utilized for anti-inflammatory and antidepressant activities evaluation were purchased from Pfizer Laboratories (Eljadidam, Morocco). Five standard organic acids, including malic, citric, oxalic, fumaric, and L-ascorbic acids, were obtained from Sigma-Aldrich (Munich, Germany). Chlorogenic acid, gallic acid, caffeic acid, myricetin, and naringenin were purchased from Sigma-Aldrich (St. Louis, MO, USA).

### 3.2. Preparation of Apple Vinegar

Four Moroccan apple cultivars—Golden Delicious, Red Delicious, Gala, and Starking Delicious—were used in this study. The varieties were collected in September 2019 from the Imouzzer region (33°45′40.9″ N; 5°00′44.8″ W) in Morocco. The vinegar of each cultivar was produced from 500 g of apples (mixture of apples at varying degrees of maturity) using the traditional process at room temperature (22 ± 3 °C) and sheltered from light [3]. The alcoholic fermentation of all samples of apple was performed in hermetically sealed glass bottles for 45 days. The fermented mash obtained, including small pieces of apples, was filtered under pressure for acetic fermentation with aerobic conditions for 20 days at room temperature (22 ± 3 °C). Three replicates of each type of vinegar were carried out under the same conditions, and the produced vinegar was stored at 6 °C until analysis.

### 3.3. Physicochemical Properties

The pH of vinegar samples was measured using a calibrated Bench Meter pH-210 (Hanna Instruments, Washington, DC, USA), while the density (g/cm^3^) of the samples was measured using a density meter instrument and the electric conductivity (mS/cm) was measured using a bench-top conductivity meter HI2315-01 (Hanna Instruments, Smithfield, RI, USA). Also, the acidity of the samples was determined via titration with NaOH (0.1 mol/L) as a percentage of acetic acid equivalent [60].

### 3.4. Total Phenolic and Flavonoid Contents

The total phenolic content (TPC) was assessed using the Folin–Ciocalteu method [24]. The quantification was expressed as mg of gallic acid equivalent per gram of vinegar (mg GAE/g). Conversely, the total flavonoid content (TFC) was quantified according to the modified aluminum trichloride method described previously by [17]. The results were calculated based on a calibration curve of quercetin and expressed as mg of quercetin equivalent per gram of vinegar. All tests were carried out in triplicate.

### 3.5. HPLC Analysis of Organic Acids

Organic acid contents, including malic, citric, oxalic, fumaric, and L-ascorbic, were determined using High-Performance Liquid Chromatography (HPLC), as described by [4] with slight modifications. The extraction of organic acids was carried out by mixing 1 mL of the sample with 4 mL of ultra-distilled water. The mixture was sonicated in an ultrasonic water bath at a temperature of 80 °C for 15 min and then centrifuged at 5500 rpm. Subsequently, filtration of the mixture was performed using a Whatman nylon syringe filter (0.45 µm, 13.00 mm/diameter), and the extract of organic acids was analyzed using an HPLC (Shimadzu LC 20A VP, Kyoto, Japan) coupled with a UV detector (Shimadzu SPD 20A VP, Kyoto, Japan) and an 87 H column (5.00 µm, 300.00 mm × 7.80 mm inside diameter, Transgenomic, Barcelona, Spain). Noteworthy, the volume injected was 20 µL; the detection wavelength was 210 nm and 242 nm, the flow rate was 0.8 mL/min, and sulfuric acid (0.05 mM) was used as a mobile phase solvent. Furthermore, the column temperature was set at 40 °C. The organic acids were identified based on the retention times and comparison of spectral data with the standards. The quantification of these organic acids was carried out using the standard calibration curves. The results obtained were expressed as mg/100 g.

### 3.6. HPLC-DAD Analysis of Polyphenols

Quantification of phenolic compounds such as caffeic acid, gallic acid, chlorogenic acid, myricetin, and naringenin was determined using High-Performance Liquid Chromatography coupled with a UV diode array detector (HPLC-DAD) following the procedure described by [61]. A total of 1 mL of each vinegar sample was mixed with 5 mL of methanol/water (80:20, *v*/*v*). After manual shaking for 1 min, the extraction was conducted in an ultrasonic bath at room temperature for 15 min. Then, the mixture was centrifuged at 5000 rpm for 25 min, and an aliquot of the supernatant phase was filtered through a 5 mL plastic syringe with a 0.45 µm PVDF filter prior to analysis. HPLC analysis of phenolic compounds was carried out using an Agilent 1100 system (Agilent Technology, Urdorf, Switzerland) with a diode array detector (DAD). The system consisted of a quaternary pump, a degasser, and an automatic sampler. A Nucleosil 100 C18 column (250 × 4.6 mm, 5 µm) was used for HPLC separation. The injection volume was 20 µL, the temperature of the column was set at 30 °C, and the flaw rate was set at 1.5 mL/min. The mobile phase consisted of two solvents: Solvent A, water/acetic acid (99.9:0.1; *v/v*), and Solvent B, acetonitrile/acetic acid A (99.9:0,1; *v/v*). The phenolic compounds were detected at 280 nm and 320 nm, and the results obtained were expressed as µg/g of vinegar.

### 3.7. In Vitro Antioxidant Activity

The antioxidant capacity of vinegar was assessed by three tests, including DPPH radical scavenging, β-carotene discoloration, and total antioxidant capacity (TAC).

#### 3.7.1. DPPH Free Radical Scavenging Assay

The DPPH test was carried out following the protocol described by [17]. A total of 100 µL of each vinegar sample at different concentrations was combined with 750 µL of 2,2, diphenyl-1-picrylhydrazyl (DPPH) solution previously prepared in methanol (100 µM). After 30 min of incubation at room temperature, the solution’s absorbance was measured at 517 nm in comparison with the negative control. Butylated hydroxytoluene (BHT) was used as a standard antioxidant in the same test. The formula below was used to calculate the percentage of DPPH radical inhibition by vinegar samples:

DPPH inhibition (%) = [1 − (Abs/Abs_0_)] × 100

Abs and Abs_0_ represent the DPPH solution’s absorbances in the presence and the absence (negative control) of the vinegar, respectively.

The antioxidant capacity of the vinegar studied was interpreted from their IC_50_, expressed in mg/mL.

#### 3.7.2. β-Carotene Discoloration Assay

In this test, 10 µL of linoleic acid was added to 100 mL of Tween 80 and mixed with 1 mL of β-carotene solution previously prepared in chloroform (2 mg/10 mL). The chloroform solvent was vacuum-evaporated at 45 °C. Then, 25 mL of hydrogen peroxide was added gradually to the residue, and the resulting mixture was stirred vigorously to obtain a stable emulsion. A total of 100 µL of each sample was mixed with 2.5 mL of the previously prepared mixture. After incubation in a water bath at 50 °C, the solutions’ absorbance was measured at 470 nm at 0 min and after 120 min against a blank without β-carotene [62]. Under the same conditions, distilled water and BHT were also used as controls.

The antioxidant activity (AA) of the vinegar samples was expressed as percent inhibition of β-carotene discoloration using the following formula:

AA (%) = [1 − (A0 − At)/(Ac0 − Act)] × 100

A0 and Ac0 represent the absorbances at t = 0 min of incubation for the sample and negative control, respectively. At and Act are the absorbances measured after t = 120 min of incubation for the sample and negative control, respectively.

#### 3.7.3. Total Antioxidant Capacity (TAC) Assay

A total of 25 mL of each vinegar sample was combined with 1 mL of the reagent solution (28 mM sodium phosphate, 0.6 M sulfuric acid, and 4 mM ammonium molybdate). The mixture was incubated for 90 min at 95 °C, and the absorbance was measured at 695 nm in comparison to a blank [63]. The total antioxidant capacity was measured using the ascorbic acid calibration curve and expressed as milligrams of ascorbic acid equivalent per gram of vinegar (mg AAE/g of vinegar). Quercetin was used as a standard antioxidant following the same protocol. The test was performed in triplicate.

### 3.8. Antimicrobial Activity

#### 3.8.1. Growth Medium and Chemicals

Müller–Hinton Broth (MHB) and Müller–Hinton Agar (MHA) were used for the bacteria strains. Sabouraud (SB) and Yeast Peptone Glucose (YPG) were used for the fungal strain [64]. Moreover, all mediums were prepared and autoclaved for 20 min at 120 °C [65]. Ampicillin (AMP) was used against the bacterial strains, and fluconazole (FLU) was used against the fungal strain. Antibiotics and antifungals were sterilized via filtration.

#### 3.8.2. Microbial Strains

The antimicrobial activity of vinegar was studied against four microorganisms. These microbial pathogens were deposited in the American Type Culture Collection (ATCC). Three bacteria strains were used: *Escherichia coli* ATCC 25922 (Gram-negative), *Bacillus subtilis* ATCC 6633 (Gram-positive), and *Staphylococcus aureus* ATCC 29213 (Gram-positive), and one yeast strain: *Candida albicans* ATCC 10231. The microbial strains tested were provided by the Microbiology Laboratory, Faculty of Medicine and Pharmacy of Fez, Morocco. The purity of the strains was verified by macroscopic characterization (colony characteristics) as well as by microscopic observation of smears stained with Gram staining and by microculture for the *Candida albicans* strain. Subsequently, the microbial strains were preserved in a solution of glycerol (25%) and MHB medium (75%), then stored in a freezer at −80 °C.

#### 3.8.3. Preparation of the Microbial Suspensions

The microbial suspension was prepared by directly picking two to three colonies from a fresh 24-h-old culture, which was collected aseptically and suspended in 0.9% NaCl solution. Then, the optical density of the suspensions was verified with a UV-Visible spectrophotometer (Selecta, E. U) at the wavelength λ = 625 nm. Therefore, the yeast suspension contains approximately 1–5 × 10^6^ CFU/mL, whereas the bacterial suspensions contain approximately 1–2 × 10^8^ CFU/mL at an absorbance of 0.08–0.13 [65].

#### 3.8.4. Agar Well Diffusion Assay

The microbial strains’ sensitivity was tested by the agar well diffusion method frequently used to assess antimicrobial activity [62]. First, 1 mL of fresh microbial culture was added to Petri dishes (90 mm) containing MHA and SB medium. Next, five perforations of 6 mm were made on the agar surface, including the positive controls. Then, 50 µL of sterile filtered vinegar samples and the positive controls (AMP: 0.5 mg/mL, FLU: 5 mg/mL) was deposed in each well. Finally, the Petri dishes were incubated at 37 °C for the bacterial strains and 30 °C for the yeast for 24 h. After incubation, the zones around the wells were measured in mm. The test was repeated three times to ensure reliability [66].

#### 3.8.5. Determination of the Minimal Inhibitory Concentration (MIC)

In this test, the microdilution method was used to evaluate the MICs [63]. Serial dilutions of the vinegar samples were prepared directly in a 96-well microplate containing MHB and YPG broth to obtain different concentrations. First, 50 μL of the growth medium was deposited in all the wells of the microplate. Then, 100 μL of each vinegar sample was added to the first well. After that, a microdilution was performed by transferring a volume of 50 μL from the first well to the second, and so on (diluting the material by a factor of ½ in each well), except for the last well (positive growth control). Next, the inoculation was carried out by depositing 50 μL of the microbial suspension in all the wells except for the negative growth control. Finally, the incubation of the microplate was conducted for 24 h at a temperature of 37 °C for the bacterial strains and at 30 °C for the yeast strain. After incubation and before reading the results, a volume of 20 μL of (1%) 2,3,5-triphenyl tetrazolium chloride (TTC) was added to each well. Indeed, the wells with bacterial growth turned pink under the effect of the dehydrogenases, whereas the wells without bacterial growth remained colorless after 2 h of incubation. Therefore, the MIC reflects the lowest concentration that inhibits the growth of microorganisms.

### 3.9. Animal Handling and Housing

Adult male and female Wistar rats weighing 130–190 g (anti-inflammatory test) and 110–180 g (antidepressant test) were acquired from the Emirates Wildlife Propagation Center in Missour, Morocco. Animals were housed in cages (5 rats/cage) in a controlled environment with temperature maintained at 22.0 ± 2.0 °C, 55.0 ± 5.0% humidity, and a light/dark cycle of 12:12 h for a 2-week acclimatization period. The rats had free access to food and water throughout the study period. All activities and procedures of this study were conducted in accordance with the internationally accepted standard guidelines of the European community for the protection of animals used for experimental and other scientific purposes (council directive of 24 November 1986). The rats used were kept alive after the tests with free access to water and food.

### 3.10. Anti-Inflammatory Test

The carrageenan-induced rat paw inflammation test was performed to evaluate the anti-inflammatory activity of vinegar samples following the procedure described by [67]. Wistar rats were divided into 6 groups (*n* = 5). Group 1 represented the negative control and received only NaCl (0.9%). Regarding the second to the fifth group, each received orally one of the four vinegars studied (diluted 3 times because of their high acidity) at a dose of 10 mL/Kg. The sixth group received diclofenac (1%) as a positive control. One hour later, the circumference of the right paw of each group was measured before injection of carrageenan (1%); then, edema measurements were continued after 3,4,5, 6, and 24 h of injection. The severity of the edema was assessed by determining the mean percentage increase in rat paw circumference according to the formula below:

Oedema (%) = [(C_t_ − C_0_)/C_0_] × 100

C_0_ and C_t_ represent the initial circumference and the circumference of the paw at time t, respectively.

The anti-inflammatory activity of the studied samples was also evaluated by calculating the inhibition of inflammation according to the following equation:

Inflammation inhibition (%) = [(% Oedema (C) − % Oedema (T))/% Oedema (C)] × 100

Edema (C) and Edema (T) correspond to the percent increases in edema observed in the control and treated rat groups, respectively.

### 3.11. Antidepressant Activity

The forced swimming test was conducted to evaluate antidepressant activity according to the protocol described by [68]. Six groups of Wistar rats were formed (*n* = 5). Group 1 (negative control) received NaCl (0.9%). The four groups from the second to the fifth each received orally one of the four kinds of vinegar studied at a dose of 10 mL/Kg. The last group received sertraline at a dose of 10 mg/kg. A total of 30 min after the administration of the vinegar, the rats were forced to swim for 8 min in a vertical glass cylinder (40 cm high, 31 cm in diameter) containing fresh water (23 °C) to a level that prevents the rat’s tail from touching the bottom. Immobility time (during which animals float in the water in an upright position and make only small movements to keep their heads above water and avoid drowning) was recorded manually during the last 6 min of the test.

### 3.12. Statistical Analysis

Data of this study were expressed as mean and standard deviation (SD) or standard error of the mean (SEM). ANOVA followed by Tukey’s multiple range test was performed to assess the significance of the differences and to compare the means of various treatment groups. The results were considered statistically significant when *p* < 0.05. Pearson’s correlation coefficient (r) at 99% significance level (*p* < 0.01) and PCA statistical analyses were accomplished using GraphPad Prism 9.4.1 (Microsoft Software; Los Angeles, CA, USA).

## 4. Conclusions

This study provides new information concerning the impact of processing various Moroccan apple cultivars (Golden Delicious, Red Delicious, Starking Delicious, and Gala) on the composition and biological properties of vinegar. Additionally, this research provides novel findings regarding the antidepressant and anti-inflammatory activities exhibited by the studied vinegars. The apple variety seems to influence the total acidity, aromatic volatiles, polyphenolic, and organic acids profile of vinegar. Therefore, the organoleptic proprieties and the biological and pharmacological activities of this vinegar were also dependent on the raw material. Golden Delicious vinegar exhibited significant anti-inflammatory and antidepressant effects. The same vinegar displayed remarkable antimicrobial activities. In contrast, Red Delicious and Gala vinegars were, in particular, more involved in inhibiting lipid oxidation due to their phenolic content. Based on the results of this study, it is recommended that apple cultivar selection be adopted to reduce the effects of chemical composition variability and to specify the desired organoleptic and biological qualities of apple vinegar. Conclusively, the vinegar studied can be considered a good natural source of bioactive molecules, especially organic acids and polyphenols, which certainly have multiple nutritional and health benefits and can be adopted in combination to combat many public health challenges, including antimicrobial resistance.

## Figures and Tables

**Table 1:** Physicochemical properties and total phenolic and flavonoid contents of studied vinegars (means ± SEM).

Kinds of Vinegar	Acetic Acid (%)	Density (g/cm^3^)	pH	Conductivity (mS/cm)	Total Phenols (mg GAE/g)	Flavonoids (mg QE/g)
Golden Delicious	2.56 ± 0.14 ^a^	1.018	3.11 ± 0.02 ^b^	3.33 ± 0.01 ^b^	3.72 ± 0.15 ^c^	0.294 ± 0.009 ^a^
Red Delicious	1.54 ± 0.07 ^b^	1.002	3.10 ± 0.02 ^b^	3.48 ± 0.02 ^a^	5.78 ± 0.08 ^a^	0.254 ± 0.008 ^a^
Gala	0.74 ± 0.09 ^c^	1.014	2.84 ± 0.01 ^c^	2.96 ± 0.02 ^d^	4.35 ± 0.02 ^b^	0.269 ± 0.010 ^a^
Starking Delicious	0.28 ± 0.06 ^d^	0.962	3.82 ± 0.04 ^a^	3.19 ± 0.02 ^c^	6.12 ± 0.02 ^a^	0.296 ± 0.012 ^a^

In each column, values with different letters are significantly different (*p* < 0.05).

**Table 2:** Polyphenols (µg/g) and organic acids (mg/100 g) of the studied apple vinegar (means ± SD). GD: Golden Delicious; RD: Red Delicious; GA: Gala; SD: Starking Delicious.

Chemical Class	Compounds	Vinegar			
		GD	RD	GA	SD
Organic acids (mg/100 g)	L-Ascorbic acid	15.78 ± 1.10 ^a^	15.33 ± 1.27 ^a^	10.94 ± 1.12 ^b^	7.78 ± 0.20 ^c^
	Oxalic acid	38.41 ± 2.10 ^b^	45.92 ± 2.11 ^a^	30.01 ± 3.10 ^c^	9.91 ± 1.08 ^d^
	Citric acid	193.63 ± 12.23 ^c^	820.62 ± 20.31 ^a^	246.50 ± 22.30 ^c^	532.86 ± 31.41 ^b^
	Malic acid	241.10 ± 10.40 ^b^	280.16 ± 11.10 ^a^	181.25 ± 10.01 ^c^	174.57 ± 9.11 ^c^
	Fumaric acid	0.39 ± 0.10 ^d^	39.74 ± 5.02 ^b^	67.32 ± 7.11 ^a^	23.01 ± 4.00 ^c^
Polyphenols (µg/g)	Gallic acid	54.40 ± 4.20 ^d^	157.37 ± 10.10 ^b^	285.70 ± 14.10 ^a^	110.81 ± 9.11 ^c^
	Caffeic acid	6.44 ± 0.31 ^a^	4.17 ± 0.21 ^b^	-	-
	Chlorogenic acid	2.65 ± 0.11 ^a^	-	-	-
	Myricetin	15.42 ± 1.41 ^b^	22.24 ± 2.12 ^a^	-	-
	Naringenin	-	-	-	1.84 ± 0.21 ^a^

In each row, values with different letters are significantly different (*p* < 0.05).

**Table 3:** Antioxidant activities of vinegar studied (means ± SD).

Samples	DPPH (IC_50_ mg/mL)	β-Carotene Antiradical Activity (%)	TAC (mg AAE/g)
Golden Delicious	596.00 ± 29.50 ^e^	62.50 ± 2.40 ^d^	26.45 ± 0.82 ^c^
Red Delicious	65.20 ± 13.00 ^b^	81.67 ± 3.02 ^b^	25.64 ± 0.36 ^c^
Gala	358.60 ± 26.30 ^d^	85.83 ± 2.13 ^ab^	16.58 ± 0.18 ^e^
Starking Delicious	187.70 ± 18.10 ^c^	69.17 ± 2.03 ^c^	18.73 ± 0.34 ^d^
BHT	0.15 ± 0.04 ^a^	91.06 ± 0.82 ^a^	46.87 ± 0.72 ^a^
Quercetin	-	-	28.06 ± 0.29 ^b^

For each test, values with different letters in each column are significantly different (*p* < 0.05).

**Table 4:** Inhibition diameter (means ± SD) of vinegar tested against bacterial and fungal strains.

Inhibition Diameter (mm)				
Vinegars	Yeast	Gram (−) Bacteria	Gram (+) Bacteria	
	*C. albicans*	*E. coli*	*B. subtilis*	*S. aureus*
Golden Delicious	11.50 ± 1.50 ^b^	15.00 ± 1.00 ^b^	25.00 ± 3.00 ^b^	14.50 ± 0.50 ^b^
Red Delicious	7.50 ± 0.50 ^c^	12.00 ± 3.00 ^b^	10.00 ± 2.00 ^c^	8.50 ± 1.50 ^d^
Gala	7.75 ± 0.25 ^c^	10.50 ± 2.50 ^b^	17.00 ± 2.00 ^c^	10.75 ± 0.25 ^c^
Starking Delicious	7.25 ± 0.75 ^c^	Resistant	12.00 ± 4.00 ^c^	Resistant
AMP	-	34.67 ± 2.08 ^a^	53.83 ± 1.04 ^a^	34.17 ± 0.28 ^a^
FLU	21.00 ± 1.04 ^a^	-	-	-

In each column, values with different letters are significantly different (*p* < 0.05).

**Table 5:** MIC results of vinegar tested against bacterial and fungal strains.

Minimal Inhibitory Concentration (mg/mL)				
Vinegar	Yeast	Gram (−) Bacteria	Gram (+) Bacteria	
	*C. albicans*	*E. coli*	*B. subtilis*	*S. aureus*
Golden Delicious	31.81	127.25	31.81	31.81
Red Delicious	125.25	62.62	62.62	62.62
Gala	126.75	126.75	126.75	253.5
Starking Delicious	481	Resistant	481	Resistant
AMP	-	Resistant	Resistant	Resistant
FLU	0.4	-	-	-

**Table 6:** Pearson correlation coefficients (r; *p* < 0.05) between different parameters and biological activities of apple vinegar.

	DPPH (IC50)	β-Carotene	TAC	*C. albicans* (ID) *	*E. coli* (ID)	*B. subtilis* (ID)	*S. aureus* (ID)	Anti-Inflammatory Activity (24 h)	Antidepressant Activity (%)
Phenols	−0.919	0.205	−0.177	−0.802	−0.731	−0.930	−0.885	−0.788	−0.315
Flavonoids	0.558	−0.852	−0.086	0.459	−0.382	0.508	−0.206	0.111	0.182
pH	−0.323	−0.515	−0.133	−0.266	−0.848	−0.361	−0.853	−0.508	−0.045
Acetic acid	0.577	−0.439	0.867	0.868	0.851	0.669	0.812	0.976	0.903

* ID = Inhibition diameter.

## Data Availability

The data presented in this study are available upon request.

## References

[1] Radenkovs V., Püssa T., Juhnevica-Radenkova K., Kviesis J., Salar F.J., Moreno D.A., Drudze I. (2020). Wild Apple (*Malus* spp.) by-Products as a Source of Phenolic Compounds and Vitamin C for Food Applications. Food Biosci..

[2] Han M.L., Zhao Y.H., Meng J.X., Yin J., Li H.H. (2023). Analysis of Physicochemical and Antioxidant Properties of *Malus* spp. Petals Reveals Factors Involved in Flower Color Change and Market Value. Sci. Hortic..

[3] Kara M., Assouguem A., Al Kamaly O.M., Benmessaoud S., Imtara H., Mechchate H., Hano C., Zerhouni A.R., Bahhou J. (2021). The Impact of Apple Variety and the Production Methods on the Antibacterial Activity of Vinegar Samples. Molecules.

[4] Liu Q., Tang G.Y., Zhao C.N., Gan R.Y., Li H. (2019). Bin Antioxidant Activities, Phenolic Profiles, and Organic Acid Contents of Fruit Vinegars. Antioxidants.

[5] Wang X., He L., Huang Z., Zhao Q., Fan J., Tian Y., Huang A. (2023). Isolation, Identification and Characterization of a Novel Antimicrobial Peptide from Moringa Oleifera Seeds Based on Affinity Adsorption. Food Chem..

[6] Yagnik D., Serafin V., Shah A.J. (2018). Antimicrobial Activity of Apple Cider Vinegar against *Escherichia coli*, *Staphylococcus aureus* and *Candida albicans*; Downregulating Cytokine and Microbial Protein Expression. Sci. Rep..

[7] Pianta L., Vinciguerra A., Bertazzoni G., Morello R., Mangiatordi F., Lund V.J., Trimarchi M. (2020). Acetic Acid Disinfection as a Potential Adjunctive Therapy for Non-Severe COVID-19. Eur. Arch. Oto-Rhino-Laryngol..

[8] Gaber S.N., Bassyouni R.H., Masoud M., Ahmed F.A. (2020). Promising Anti-Microbial Effect of Apple Vinegar as a Natural Decolonizing Agent in Healthcare Workers. Alex. J. Med..

[9] Baldas B., Altuner E.M. (2018). The Antimicrobial Activity of Apple Cider Vinegar and Grape Vinegar, Which Are Used as a Traditional Surface Disinfectant for Fruits and Vegetables. Commun. Fac. Sci. Univ. Ankara Ser. C Biol..

[10] Rutala W.A., Barbee S.L., Aguiar N.C., Sobsey M.D., Weber D.J. (2000). Antimicrobial Activity of Home Disinfectants and Natural Products Against Potential Human Pathogens. Infect. Control Hosp. Epidemiol..

[11] Zhang H., Zhu X., Huang Q., Zhang L., Liu X., Liu R., Lu Q. (2023). Antioxidant and Anti-Inflammatory Activities of Rape Bee Pollen after Fermentation and Their Correlation with Chemical Components by Ultra-Performance Liquid Chromatography-Quadrupole Time of Flight Mass Spectrometry-Based Untargeted Metabolomics. Food Chem..

[12] Bucciantini M., Leri M., Nardiello P., Casamenti F., Stefani M. (2021). Olive Polyphenols: Antioxidant and Anti-Inflammatory Properties. Antioxidants.

[13] Ousaaid D., Imtara H., Laaroussi H., Lyoussi B., Elarabi I. (2021). An Investigation of Moroccan Vinegars: Their Physicochemical Properties and Antioxidant and Antibacterial Activities. J. Food Qual..

[14] Alberti A., Machado dos Santos T.P., Ferreira Zielinski A.A., Eleutério dos Santos C.M., Braga C.M., Demiate I.M., Nogueira A. (2016). Impact on Chemical Profile in Apple Juice and Cider Made from Unripe, Ripe and Senescent Dessert Varieties. LWT-Food Sci. Technol..

[15] Ousaaid D., Laaroussi H., Bakour M., ElGhouizi A., Aboulghazi A., Lyoussi B., ElArabi I. (2020). Beneficial Effects of Apple Vinegar on Hyperglycemia and Hyperlipidemia in Hypercaloric-Fed Rats. J. Diabetes Res..

[16] Tripathi S., Mazumder P.M. (2020). Apple Cider Vinegar (ACV) and Their Pharmacological Approach towards Alzheimer’s Disease (AD): A Review. Indian J. Pharm. Educ. Res..

[17] Ozturk I., Caliskan O., Tornuk F., Ozcan N., Yalcin H., Baslar M., Sagdic O. (2015). Antioxidant, Antimicrobial, Mineral, Volatile, Physicochemical and Microbiological Characteristics of Traditional Home-Made Turkish Vinegars. LWT-Food Sci. Technol..

[18] Bogdanov S., Jurendic T., Sieber R., Gallmann P. (2008). Honey for Nutrition and Health: A Review. J. Am. Coll. Nutr..

[19] Hecke K., Herbinger K., Veberič R., Trobec M., Toplak H., Štampar F., Keppel H., Grill D. (2006). Sugar-, Acid- and Phenol Contents in Apple Cultivars from Organic and Integrated Fruit Cultivation. Eur. J. Clin. Nutr..

[20] Ergün Z. (2021). Determination of Biochemical Contents of Fresh, Oven-Dried, and Sun-Dried Peels and Pulps of Five Apple Cultivars (Amasya, Braeburn, Golden Delicious, Granny Smith, and Starking). J. Food Qual..

[21] Duan W., Xia T., Zhang B., Li S., Zhang C., Zhao C., Song J., Wang M. (2019). Changes of Physicochemical, Bioactive Compounds and Antioxidant Capacity during the Brewing Process of Zhenjiang Aromatic Vinegar. Molecules.

[22] Zhang S., Hu C., Guo Y., Wang X., Meng Y. (2021). Polyphenols in Fermented Apple Juice: Beneficial Effects on Human Health. J. Funct. Foods.

[23] Ye M., Yue T., Yuan Y. (2014). Evolution of Polyphenols and Organic Acids during the Fermentation of Apple Cider. J. Sci. Food Agric..

[24] Kahraman H.A., Tutun H., Keyvan E., Balkan B.M. (2022). Bioactive Components, Antibacterial and Antiradical Properties of Home-Made Apple and Grape Vinegar. Ank. Üniversitesi Vet. Fakültesi Derg..

[25] Wei J., Zhang Y., Wang Y., Ju H., Niu C., Song Z., Yuan Y., Yue T. (2020). Assessment of Chemical Composition and Sensorial Properties of Ciders Fermented with Different Non-Saccharomyces Yeasts in Pure and Mixed Fermentations. Int. J. Food Microbiol..

[26] Ren M., Wang X., Tian C., Li X., Zhang B., Song X., Zhang J. (2017). Characterization of Organic Acids and Phenolic Compounds of Cereal Vinegars and Fruit Vinegars in China. J. Food Process. Preserv..

[27] Liu Q., Tang G.Y., Zhao C.N., Feng X.L., Xu X.Y., Cao S.Y., Meng X., Li S., Gan R.Y., Li H. (2018). Bin Comparison of Antioxidant Activities of Different Grape Varieties. Molecules.

[28] Ho C.W., Lazim A.M., Fazry S., Zaki U.K.H.H., Lim S.J. (2017). Varieties, Production, Composition and Health Benefits of Vinegars: A Review. Food Chem..

[29] Denis M.C., Furtos A., Dudonné S., Montoudis A., Garofalo C., Desjardins Y., Delvin E., Levy E. (2013). Apple Peel Polyphenols and Their Beneficial Actions on Oxidative Stress and Inflammation. PLoS ONE.

[30] Pathak L., Agrawal Y., Dhir A. (2013). Natural Polyphenols in the Management of Major Depression. Expert Opin. Investig. Drugs.

[31] Seydim A.C., Guzel-Seydim Z.B., Doguc D.K., Savas M.C., Budak H.N. (2016). Effects of Grape Wine and Apple Cider Vinegar on Oxidative and Antioxidative Status in High Cholesterol-Fed Rats. Funct. Foods Health Dis..

[32] EL Moussaoui A., Bourhia M., Jawhari F.Z., Salamatullah A.M., Ullah R., Bari A., Majid Mahmood H., Sohaib M., Serhii B., Rozhenko A. (2021). Chemical Profiling, Antioxidant, and Antimicrobial Activity against Drug-Resistant Microbes of Essential Oil from *Withania frutescens* L. Appl. Sci..

[33] El-Sayed T.S., Nour El-Deen M.M., Rokaya M.E., Sherif M.M. (2019). Evaluation of the Antibacterial Effect of Apple Vinegar as a Root Canal Irrigant Using Endovac Irrigation System. Al-Azhar Dent. J. Girls.

[34] El Atki Y., Aouam I., El Kamari F., Taroq A., Lyoussi B., Oumokhtar B., Abdellaoui A. (2020). Phytochemistry, Antioxidant and Antibacterial Activities of Two Moroccan Teucrium Polium L. Subspecies: Preventive Approach against Nosocomial Infections. Arab. J. Chem..

[35] Hirshfield I.N., Terzulli S., O’Byrne C. (2003). Weak Organic Acids: A Panoply of Effects on Bacteria. Sci. Prog..

[36] Salmond C.V., Kroll R.G., Booth I.R. (1984). The Effect of Food Preservatives on PH Homeostasis in *Escherichia coli*. J. Gen. Microbiol..

[37] Bjornsdottir K., Breidt F., McFeeters R.F. (2006). Protective Effects of Organic Acids Oil Survival of *Escherichia coli* O157:H7 in Acidic Environments. Appl. Environ. Microbiol..

[38] Chen H., Chen T., Giudici P., Chen F. (2016). Vinegar Functions on Health: Constituents, Sources, and Formation Mechanisms. Compr. Rev. Food Sci. Food Saf..

[39] Kelebek H., Kadiroğlu P., Demircan N.B., Selli S. (2017). Screening of Bioactive Components in Grape and Apple Vinegars: Antioxidant and Antimicrobial Potential. J. Inst. Brew..

[40] Lima V.N., Oliveira-Tintino C.D.M., Santos E.S., Morais L.P., Tintino S.R., Freitas T.S., Geraldo Y.S., Pereira R.L.S., Cruz R.P., Menezes I.R.A. (2016). Antimicrobial and Enhancement of the Antibiotic Activity by Phenolic Compounds: Gallic Acid, Caffeic Acid and Pyrogallol. Microb. Pathog..

[41] Medina E., Romero C., Brenes M., De Castro A. (2007). Antimicrobial Activity of Olive Oil, Vinegar, and Various Beverages against Foodborne Pathogens. J. Food Prot..

[42] Pinto T.M.S., Neves A.C.C., Leão M.V.P., Jorge A.O.C. (2008). Vinegar as an Antimicrobial Agent for Control of Candida Spp. in Complete Denture Wearers. J. Appl. Oral Sci..

[43] Tekulu G.H., Hiluf T., Brhanu H., Araya E.M., Bitew H., Haile T. (2020). Anti-Inflammatory and Anti-Nociceptive Property of Capparis Tomentosa Lam. Root Extracts. J. Ethnopharmacol..

[44] Nwidu L.L., Airhihen B., Ahmadu A. (2016). Anti-Inflammatory and Anti-Nociceptive Activities of Stem-Bark Extracts and Fractions of *Carpolobia lutea* (*Polygalaceae*). J. Basic Clin. Pharm..

[45] Murthuza S., Manjunatha B.K. (2018). In Vitro and In Vivo Evaluation of Anti-Inflammatory Potency of *Mesua ferrea*, *Saraca asoca*, *Viscum album* & *Anthocephalus cadamba* in Murine Macrophages Raw 264.7 Cell Lines and Wistar Albino Rats. Beni-Suef Univ. J. Basic Appl. Sci..

[46] Adnan M., Nazim Uddin Chy M., Mostafa Kamal A.T.M., Barlow J.W., Faruque M.O., Yang X., Uddin S.B. (2019). Evaluation of Anti-Nociceptive and Anti-Inflammatory Activities of the Methanol Extract of *Holigarna caustica* (Dennst.) Oken Leaves. J. Ethnopharmacol..

[47] Jing L., Yanyan Z., Junfeng F. (2015). Acetic Acid in Aged Vinegar Affects Molecular Targets for Thrombus Disease Management. Food Funct..

[48] Asejeje F.O., Abiola M.A., Ben-Azu B., Adeosun A.M., Ighodaro O.M., Ajayi A.M. (2020). Apple Cider Vinegar Attenuates Lipopolysaccharide-Induced Neurobehavioral Deficits in Mice. Nutrire.

[49] Shen F., Feng J., Wang X., Qi Z., Shi X., An Y., Zhang Q., Wang C., Liu M., Liu B. (2016). Vinegar Treatment Prevents the Development of Murine Experimental Colitis via Inhibition of Inflammation and Apoptosis. J. Agric. Food Chem..

[50] Sureda A., Tejada S. (2015). Polyphenols and Depression: From Chemistry to Medicine. Curr. Pharm. Biotechnol..

[51] Ueda H., Yamazaki C., Yamazaki M. (2004). A Hydroxyl Group of Flavonoids Affects Oral Anti-Inflammatory Activity and Inhibition of Systemic Tumor Necrosis Factor-α Production. Biosci. Biotechnol. Biochem..

[52] Felger J.C. (2019). Role of Inflammation in Depression and Treatment Implications. Handb. Exp. Pharmacol..

[53] O’Connor J.C., Lawson M.A., André C., Moreau M., Lestage J., Castanon N., Kelley K.W., Dantzer R. (2008). Lipopolysaccharide-Induced Depressive-like Behavior Is Mediated by Indoleamine 2,3-Dioxygenase Activation in Mice. Mol. Psychiatry.

[54] Yohn C.N., Gergues M.M., Samuels B.A. (2017). The Role of 5-HT Receptors in Depression. Mol. Brain.

[55] Bayes J., Schloss J., Sibbritt D. (2020). Effects of Polyphenols in a Mediterranean Diet on Symptoms of Depression: A Systematic Literature Review. Adv. Nutr..

[56] Ali S., Corbi G., Maes M., Scapagnini G., Davinelli S. (2021). Exploring the Impact of Flavonoids on Symptoms of Depression: A Systematic Review and Meta-Analysis. Antioxidants.

[57] Khan H., Perviz S., Sureda A., Nabavi S.M., Tejada S. (2018). Current Standing of Plant Derived Flavonoids as an Antidepressant. Food Chem. Toxicol..

[58] Budak N.H., Aykin E., Seydim A.C., Greene A.K., Guzel-Seydim Z.B. (2014). Functional Properties of Vinegar. J. Food Sci..

[59] Yun J.H., Kim Y.J., Koh K.H. (2016). Investigation into Factors Influencing Antioxidant Capacity of Vinegars. Appl. Biol. Chem..

[60] Mat Isham N.K., Mokhtar N., Fazry S., Lim S.J. (2019). The Development of an Alternative Fermentation Model System for Vinegar Production. LWT.

[61] Kašpar M., Bajer T., Bajerová P., Česla P. (2022). Comparison of Phenolic Profile of Balsamic Vinegars Determined Using Liquid and Gas Chromatography Coupled with Mass Spectrometry. Molecules.

[62] El Abdali Y., Beniaich G., Mahraz A.M., El Moussaoui A., Bin Jardan Y.A., Akhazzane M., Chebaibi M., Nafidi H.-A., Eloutassi N., Bourhia M. (2023). Antibacterial, Antioxidant, and in Silico NADPH Oxidase Inhibition Studies of Essential Oils of *Lavandula dentata* against Foodborne Pathogens. Evid.-Based Complement. Altern. Med..

[63] El Abdali Y., Mahraz A.M., Beniaich G., Mssillou I., Chebaibi M., Jardan Y.A.B., Lahkimi A., Nafidi H.-A., Aboul-Soud M.A.M., Bourhia M. (2023). Essential Oils of *Origanum compactum* Benth: Chemical Characterization, In Vitro, In Silico, Antioxidant, and Antibacterial Activities. Open Chem..

[64] Agour A., Mssillou I., Saghrouchni H., Bari A., Lyoussi B., Derwich E. (2020). Chemical Composition, Antioxidant Potential and Antimicrobial Properties of the Essential Oils of *Haplophyllum tuberculatum* (Forsskal) A. Juss from Morocco. Trop. J. Nat. Prod. Res..

[65] Balouiri M., Sadiki M., Ibnsouda S.K. (2016). Methods for In Vitro Evaluating Antimicrobial Activity: A Review. J. Pharm. Anal..

[66] Chavez-Esquivel G., Cervantes-Cuevas H., Ybieta-Olvera L.F., Castañeda Briones M.T., Acosta D., Cabello J. (2021). Antimicrobial Activity of Graphite Oxide Doped with Silver against *Bacillus subtilis*, *Candida albicans*, *Escherichia coli*, and *Staphylococcus aureus* by Agar Well Diffusion Test: Synthesis and Characterization. Mater. Sci. Eng. C.

[67] Mssillou I., Agour A., Slighoua M., Chebaibi M., Amrati F.E., Alshawwa S.Z., Kamaly O.A., El Moussaoui A., Lyoussi B., Derwich E. (2022). Ointment-Based Combination of *Dittrichia viscosa* L. and *Marrubium vulgare* L. Accelerate Burn Wound Healing. Pharmaceuticals.

[68] Morozova A.Y., Zubkov E.A., Storozheva Z.I., Kekelidze Z.I., Chekhonin V.P. (2013). Effect of Ultrasonic Irradiation on the Development of Symptoms of Depression and Anxiety in Rats. Bull. Exp. Biol. Med..

